# Further considerations for placebo controls in surgical trials

**DOI:** 10.1186/s13063-023-07417-7

**Published:** 2023-06-10

**Authors:** Marion K. Campbell, David J. Beard, Jane M. Blazeby, Sian Cousins, the ASPIRE group, Ahmed Ahmed, Rasha Al-Lamee, David B. Anderson, Natalie Blencowe, Jonathan Cook, Brian H. Cuthbertson, Manuela Ferreira, Matt Gardiner, Katie Gillies, José Miola, John Norrie, Tom Pinkney, Jonathan Pugh, Maroeska Rovers, Deborah Stocken, Matt Westmore

**Affiliations:** 1 Royal College of Surgeons of England (RCSEng) Aberdeen Surgical Trials Centre; Health Services Research Unit, Health Sciences Building, University of Aberdeen, Foresterhill, Aberdeen, UK; 2 Nuffield Department of Orthopaedics, Rheumatology and Musculoskeletal Sciences; RCSEng Surgical Intervention Trials Unit; National Institute for Health and Care Research (NIHR) Oxford Biomedical Research Centre, University of Oxford, Headington, Oxford UK; 3 NIHR Bristol Biomedical Research Centre; RCSEng Bristol Surgical Trials Centre, Bristol Centre for Surgical Research, Population Health Sciences, University of Bristol, BS8 2PS Bristol, UK

## Abstract

The use of invasive placebo controls in surgical trials can be challenging. The ASPIRE guidance, published in the Lancet in 2020, provided advice for the design and conduct of surgical trials with an invasive placebo control. Based on a more recent international expert workshop in June 2022, we now provide further insights into this topic. These include the purpose and design of invasive placebo controls, patient information provision and how findings from these trials may be used to inform decision-making.

## Introduction

Placebo controls in surgical trials are controversial. The ASPIRE guidance [1, 2] published in 2020 sought to provide advice on the criteria under which the use of placebo-controlled surgical trials may be considered ethical, when they can and should be used and how best they should be designed and delivered. The ASPIRE guidance was the product of an expert-consensus workshop, held in Oxford in 2018, together with additional research, including a systematic review of placebo-controlled surgical trials [3] and the development of a framework (DITTO) for the development of invasive placebo interventions [4]; the key element postulated to be the mechanism of action/benefit for the surgical procedure.

Since 2018, there have been several placebo-controlled surgical trials published [5–7], and others designed and commenced. Aspects of the ASPIRE guidance have been included into the conduct of ongoing placebo surgical trials [8, 9]. These have provided useful additional insights into the barriers and enablers of the design and conduct of placebo-controlled surgical trials. The use of the ASPIRE guidance has now been extended to the assessment of the quality of placebo-controlled trials in surgery [10], providing further insights into good and poor design in this complex area.

To ensure continued learning in this developing field, and to ensure insights gained from the new trials were built upon, a further workshop was held in London at the Royal College of Surgeons England in June 2022. The aim of the workshop was to address topics that had been flagged within the original ASPIRE report as worthy of further consideration and research, including those related to the purpose and design of invasive placebo controls, patient information and how findings are used to inform decision-making. This manuscript presents the deliberations and conclusions of this second workshop.

## Method

### Composition of workshop members

The workshop comprised 20 individuals from a range of backgrounds. This included surgeons, clinicians, ethicists, statisticians, methodologists, health services researchers, legal scholars and representatives from funders and regulatory agencies. Workshop members included national and international representatives. The workshop included a number of the original ASPIRE group, together with those involved in the design and conduct of recent placebo-controlled surgical trials and additional perspectives, from legal and regulatory representatives.

### Topics addressed at the workshop

The six topics addressed at the workshop were selected specifically as ongoing dilemmas in the field and those that have been flagged in the original report as being worthy of further consideration, either because of complexity or incomplete guidance/understanding. The aim of the workshop was to provide further clarification on the operationalisation of these core concepts. These topics were:

1. Why use a placebo control? What is its purpose?
2. Should placebo surgical trials have two or three arms?
3. Fidelity versus risk mitigation—what is the right balance?
4. Should placebo surgical trials have enhanced consent protections?
5. Should placebo surgical trials be equalised in terms of contextual factors?
6. What should be recommended when the placebo is as effective as the active treatment already in use?

For each topic, two/three slides were presented summarising the findings from the original ASPIRE work, the ongoing dilemma and a couple of points to initiate discussion. Ground rules were established to reinforce that all voices were equally important and that a range of views were welcome. There were two dedicated post-doctoral note-takers to ensure that the breadth of discussion was captured.

### Workshop discussions

Summaries of the workshop discussions are provided under each question separately below.

1. Why use a placebo control? What is its purpose?

When considering the use of a placebo control in a surgical trial, it was reinforced that the research question should drive the choice of design, including the decision to use a placebo control.

It was accepted that a placebo trial design remains a very powerful indicator of true efficacy and effectiveness, after controlling for the strong placebo/non-specific effects of undergoing a surgical intervention, therefore potentially offering benefits over a surgery versus no surgery design (which does not control for these effects). It allows postulated questions around mechanism of action to be answered (see below). The design remains distinct from those head-to-head comparisons of the value or benefit from different accepted active treatments, be it other surgical or non-surgical treatments. Previous insights from ASPIRE had identified that where the balance of risks to benefits is deemed to be acceptable to clinicians and patients, the use of a surgical placebo is justifiable to test the efficacy/effectiveness of new and existing surgical interventions (where doubts exist over benefits and/or evidence of efficacy is lacking). Expanding these principles, it was clear from further deliberations that there are two scenarios where the use of a placebo control is particularly advantageous. These include:

- *Providing evidence for the hypothesised mechanism of the treatment effect of a surgical procedure, particularly in the early phases of the intervention pathway*. It was recognised that, as in drug trials, and as part of the overall efficacy evaluation, it is important to understand the mechanism of action of any surgical procedure. Whilst a surgery versus no surgery design remains an appropriate comparison for some evaluations, particularly in new innovations, it cannot rule out the effect of any short-term placebo effect potentially generated through the strong influence of the psychosocial context in which surgery is delivered [11], which could then dissipate with time. A placebo control, by comparison, can formally provide evidence and quantify the hypothesised mechanism of action, as well as confirm the longevity of any observed treatment effect. If the integration of placebos in surgical trials were to be introduced more routinely early in the surgical development phase, this would support the knowledge base underpinning the surgical procedures. More formal integration of the role of a placebo comparator within the IDEAL framework [12] is recommended.
- *Providing evidence for the efficacy of interventions, where doubts exist over benefits and/or evidence of efficacy is lacking, to support the use of effective interventions or potential de-implementation those ineffective*. It was recognised that, in general, a greater level of evidence is required for health providers or regulatory agencies to disinvest from, or de-implement/de-adopt, a procedure that is already in established practice [13]. Placebo-controlled surgical trials can provide this evidence. In addition to the technical requirement of having to provide sound evidence that a treatment is ineffective (or effective), there are cultural factors to be considered if de-implementation is being deliberated, which may impede the translation of trial findings into clinical practice. These include addressing lack of equipoise in the clinical community (in response to the “we know it works” sentiment), patient preferences, financial and commercial pressures and resistance that may arise in instances where there is a lack of treatment alternatives. In such circumstances, a placebo-controlled design can produce the most rigorous level of evidence to support de-implementation decisions; however, they should not be seen purely as a vehicle for de-implementation strategies. If efficacy is observed, it similarly provides the highest standard of evidence that the effect of the surgical procedure under scrutiny is real and worthwhile.

The group discussed circumstances when placebo controls should not be considered. Because of the complexity of a placebo design for surgery, this distilled down to any research question that could be answered rigorously without resorting to placebo control. Funders and research commissioners should always ask this question when reviewing placebo control proposals, despite the evidence showing that placebo-controlled trials are low risk [14]. One example of an area unsuitable for a placebo control design may be cancer.

2. Should placebo surgical trials have two or three arms?

As with the previous discussion, the underpinning principle acknowledged was that the research question should drive the choice of design. However, from a funder’s perspective the increased cost of a 3-arm design compared with a 2-arm design requires a compelling scientific justification, as well as issues related to reduced blinding of trial personnel and potential increase in trial group crossovers.

**Fig. 1:**
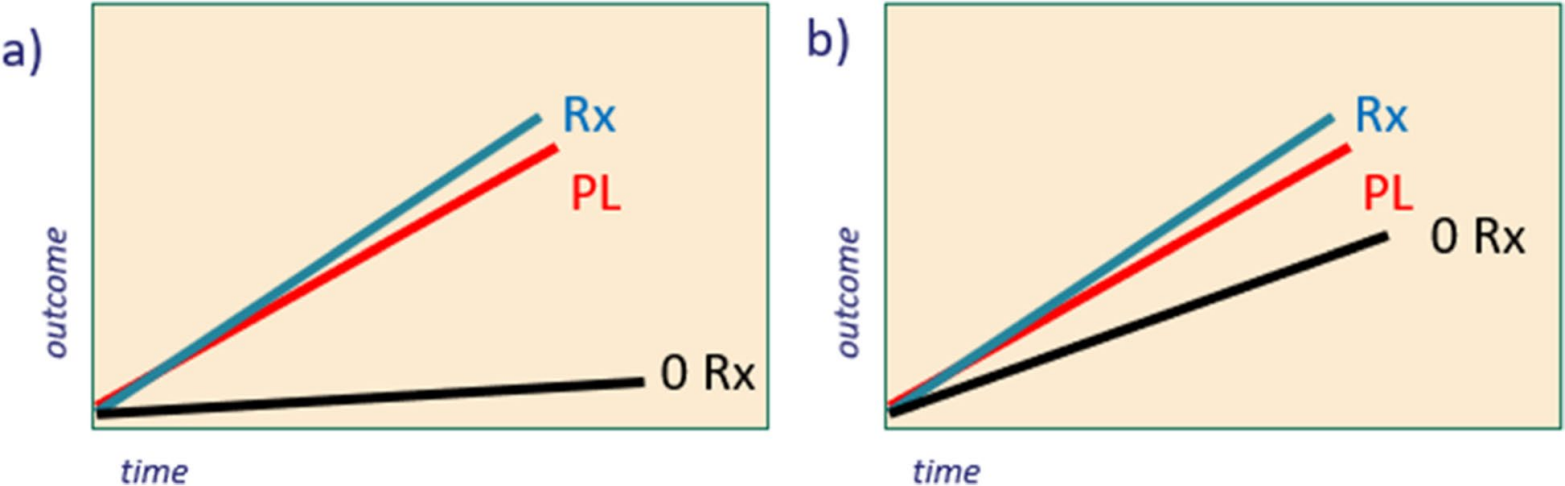
Three-arm trial of surgical intervention versus placebo versus no treatment. Rx, surgical intervention; PL, placebo; 0 Rx, no treatment. **a** Intervention and placebo are significantly more effective than no treatment. **b** Intervention no better than placebo and both no better than no treatment

There was agreement that if the social value of the experiment was purely to show that the active intervention was better than placebo, with any benefit from the placebo being disregarded, then a 2-arm trial would be sufficient. The difficulty with this scenario is that the placebo *can* (and has been shown to) provide (albeit indirectly) benefit [15] and the social value of this benefit may then be wasted or lost. Much of medicine may work on placebo models. The recent FIMPACT trial [7] demonstrated the successful utilisation of this logic. As the shoulder decompression treatment was shown to be no more beneficial than placebo, it was deemed as having no value, which can be assumed a reasonable position. However, if the research question seeks to address the efficacy of an established surgical procedure of uncertain benefits, then a 3-arm trial (surgery vs placebo vs non-operative management “do nothing”) will provide more informative data to aid subsequent decision making. For example, the scenario where the intervention and the placebo both are superior to no treatment provides very different information for decision-making compared to the scenario where the intervention (and placebo) are no more effective than doing nothing (see Fig. 1). In this case, scenario b would provide more compelling grounds for de-implementation than the different trial results profile shown in scenario a in which the no treatment arm is clearly shown to be inferior. This would stimulate a wider societal discussion about the benefit of doing something (albeit via an unknown mechanism and at significant cost) versus doing nothing—see below for further discussion. Had this model study been a 2-arm trial only, result profiles would have been identical, and the interpretation would have been the same. It was further noted that 3-arm trials may be particularly suitable when examining subjective outcomes in chronic conditions, due to the slow change in status.

3. Fidelity versus risk mitigation—what is the right balance?

In the previous work within ASPIRE [1], the concept of placebo ‘fidelity’ had been introduced—the extent to which the placebo intervention mimicked the index procedure under investigation [16] taking account of any other non-specifics effects, including placebo. In principle, one wishes to maximise the mimic of any placebo; in surgical trials, this would be to provide the cleanest and sharpest separation of the critical surgical element, that is the element believed to provide therapeutic benefit, and allow placebo effects and other non-specific effects to all be included in the same ‘section’ of the intervention. However, this needs to be traded off against minimising potential risk to both participants and a sound conclusion/interpretation of the experiment. The higher the fidelity (more intervention, more invasion), the likely higher the patient risk for peri-operative complications. The lower the fidelity, the more difficult to tease out non-specific effects, which threatens scientific validity.

There was agreement that the driving principle should be to identify the level of fidelity required to answer the scientific question and then decide whether the risk (for both patient and methodologically) associated with that level of fidelity was acceptable to allow the trial to proceed. In practice, this would equate to asking two questions: (a) what level of fidelity is necessary? and (b) is it proportionate? If the risk for the required level of fidelity is deemed to be proportionate, and transparent, unbiased and detailed information can be provided to the patient to allow truly informed consent (see section below), then use of a high-fidelity placebo control would be deemed to be acceptable. If, however, the risk was deemed not to be proportionate, then it would be unethical to continue. Similarly, if a lower level of fidelity was chosen than was required (even in an attempt to reduce risk), this would also be unethical as the placebo control would not be robust enough to ensure the scientific rigour of the trial. Consideration of these issues may be facilitated by their inclusion in existing guidance for the development of placebo invasive procedures [4], as well as wider guidance for the development and evaluation of complex interventions [17]. It was noted that patient and public involvement in intervention and placebo design is essential—patients are often open to managed risk, but full transparency and information is key. Patient and public input at the conception and study design stages will give key information about deliverability and acceptability.

4. Should placebo surgical trials have enhanced consent protections?

In our preparatory work for the original ASPIRE guidance, we had reviewed all the published international regulatory guidance on the appropriate conduct of placebo-controlled trials for surgical interventions. A number had suggested that “enhanced” consent processes be enacted for placebo surgical trials, given the non-zero risk in the placebo arm. However, there was little agreement of what enhanced consent might represent.

There was agreement that the use of the word “enhanced” was not helpful in this regard. For all trials (immaterial of the inclusion of a placebo arm), there is a legal and ethical duty to provide transparent, unbiased and comprehensive information to inform patient decision-making. As such, applying different standards of consent to placebo trials is problematic. It amplifies the incorrect notion that a placebo-controlled trial is more inherently risky than other trial designs. Core elements for inclusion in patient information leaflets were presented in the ASPIRE report. It is essential that any patient fully understands the concept, need for, nature and implication of undergoing a surgical procedure with some level of ‘placebo’ included and agrees to participate based on that understanding. This may be an avenue of further work and research.

It was further recognised by the group that there is an urgent need for wider education of both health professionals and the public of the role and nature of a placebo control and to address the stigma currently associated with the term placebo.

5. Should arms in placebo surgical trials be equalised in terms of contextual factors?

The final phase of the DITTO framework [4] for the development of a placebo surgical controls recommends the full optimisation of the placebo procedure to maximise the mimic—thus maximising blinding and minimising bias in the trial. This includes the use of components such as mimicked timings (whereby the patient spends the same amount of time in theatre as those receiving the full procedure); use of verbal cues (such as talking throughout as if the placebo were the full procedure); auditory cues (like splashing of saline) and physical cues (such as the extensive manipulation of an endoscope to simulate the full procedure). This fulsome approach to optimisation has been adopted in a number of recent placebo trials (e.g. ORBITA [6]); however, all these optimisation elements are not without additional financial cost, and also not without additional risk (e.g. due to extra time in theatre), so their use should be justified.

The group agreed that the same principles that emerged during the discussion of fidelity should also guide the approach to optimisation. This involved the trade-off between scientific rigour, risk, potential benefits, and acceptability (to patients, clinicians and healthcare systems). The fundamental principle to guide the required extent of the placebo optimisation should be the level of fidelity that is *necessary* to retain the scientific integrity of the trial. The question would then be asked whether the risk associated with that level of fidelity was *proportional*?

6. What should be recommended when the placebo is as effective as the active treatment already in use?

More than half of all placebo-controlled trials conducted in surgery report no evidence of benefit of the intervention over placebo [14]. In the original ASPIRE guidance [1], we thus outlined the need for investigators to be prepared for the results of their trial to be disruptive and to engender very mixed (sometimes uncongenial and unreceptive) responses to the results.

It is particularly contentious when the placebo itself is shown to be effective and cost-effective (Fig. 1a) and essentially fulfils the criterion laid down by national agencies (such as NICE) responsible for commissioning services. What should decision-makers do with this information? It was noted by the group that the question under consideration by commissioners at this point often shifted from ‘should we give placebo or nothing?’ to ‘should we give something or nothing?’ (whilst accepting that the mechanism of action was not that which was postulated). These discussions would require wider societal input to ensure the ethical and social consequences of these choices are fully aired and informed by inclusion of cost effectiveness analyses in placebo-controlled trials. A three-armed trial, as previously discussed, would help delineate some of the interpretation and decision making around commissioning.

From a purely scientific perspective, however, a finding of no difference between the surgical intervention and the placebo would provide strong evidence to support a call for de-implementation of that procedure as the mechanism of effect was unclear. The issue of whether replication of the placebo trial result would be required to fully action de-implementation remains unclear—as it is highly likely that the placebo result will lead to some changes to practice in any case. Replication of the (negative) result has been hugely advantageous in orthopaedic surgery to support de-implementation [7, 15].

## Conclusion and recommendations

The workshop reinforced the useful role of placebo controls in surgical trials and provided greater clarity to a number of ongoing dilemmas in their design and conduct. In particular, it highlighted the suitability of the placebo-controlled design to evaluate the hypothesised mechanism of action of a surgical procedure and to evaluate the efficacy of an existing technique but where there are doubts over its benefits. The superior benefits of adopting a 3-arm design were highlighted especially if the decision being informed included the possibility of future de-implementation. The group further concluded that the level of fidelity for the design of the placebo control should be driven by the dual principles of necessity and proportionality.

There is a clear need for wider education of both health professionals and the public of the role and nature of a placebo control in the surgical context. There was also recognition that there is stigma associated with the term placebo in this context, which should be actively addressed. Placebo as a term may not best reflect its breadth of meaning, and these interventions may be better described as controls of varying fidelity to the reference intervention accounting for placebo and non-specific effects. Further work is needed to develop meaningful, standardised and acceptable terminology.

In conclusion, if the rationale for a placebo-controlled design is justified, the required placebo control is of proportionate and acceptable risk, and consent of patients is based on transparent, unbiased and comprehensive information, the use of placebo-controlled trials in surgery can and should be promoted.

## Data Availability

Data sharing is not applicable to this article as no datasets were generated or analysed during the current study.
